# The effect of students' effort–reward imbalance on learning engagement: the mediating role of learned helplessness and the moderating role of social support

**DOI:** 10.3389/fpsyg.2024.1329664

**Published:** 2024-02-08

**Authors:** Shengmin Liu, Yuanru Wang, Wanning He, Yu Chen, Qiangqiang Wang

**Affiliations:** School of Teacher Education, Huzhou University, Huzhou, Zhejiang, China

**Keywords:** effort-reward imbalance, learning engagement, learned helplessness, social support, structural equation model

## Abstract

**Introduction:**

To explore the predictive effect of effort–reward imbalance on students' learning engagement and to elucidate the underlying mechanism, 796 students were selected for a survey.

**Methods:**

The participants were required to complete four scales: the Effort-reward Imbalance Scale, the Learning Engagement Scale, the Learned Helplessness Questionnaire, and the Perceived Social Support Scale.

**Results:**

(1) Students' effort–reward imbalance significantly and negatively predicts their learning engagement; (2) Learned helplessness serves as a mediator in the relationship between students' effort–reward imbalance and learning engagement; (3) Social support plays a moderating role in the association between effort–reward imbalance and learned helplessness. High levels of social support can buffer the impact of an effort–reward imbalance on learned helplessness, and the protective effect of social support is more obvious when the effort–reward imbalance is low.

**Discussion:**

The present study revealed how an effort–reward imbalance affects learning engagement among students through the dimensions of learned helplessness and perceived social support. The constructed model not only further clarifies the mechanism underlying the relationship between effort–reward imbalance and learning engagement but also holds significant implications for guiding students' education.

## 1 Introduction

Learning engagement is the embodiment of work engagement in the field of education. It is a lasting, universal, positive and substantial cognitive and emotional state of individual learning that is characterized by vitality, dedication and concentration (Bao et al., 2022). As a model of positive psychological traits, learning engagement has the following numerous positive characteristics: it can reflect students' positive and healthy psychological state to a certain extent; it can stimulate students' positive qualities, such as resilience, creativity and optimism; it can promote students' maturity and development; it can measure the quality of education; it can positively predict students' academic achievements; it can reflect students' development status; and it can help students successfully complete their studies (Fredricks et al., 2004; Fang et al., 2008; Schwinger et al., 2014; Liu, 2015; Wu et al., 2023). To improve students' learning engagement, scholars have conducted in-depth research on the influencing factors and underlying mechanisms of learning engagement from the aspects of academic emotions (Linnenbrink-Garcia and Pekrun, 2011; Pekrun and Linnenbrink-Garcia, 2012; Ghasemi, 2023; Liu, 2023), perceived social support (Zuo, 2022), proactive personality (Bao et al., 2022) and academic burnout (Zhang et al., 2016). For example, a study on the relationship between academic burnout and learning engagement showed that academic burnout can significantly negatively predict students' learning engagement (Duan and Li, 2008; Zhang et al., 2009; Yan et al., 2018). However, few studies have investigated the relationship between effort–reward imbalance and learning engagement from the dimensions of learned helplessness. Therefore, the present study aimed to investigate whether learned helplessness plays a mediating role in the relationship between effort–reward imbalance and learning engagement. We also aimed to test whether social support buffers the impact of an effort–reward imbalance on learning engagement by decreasing learned helplessness.

### 1.1 Effort–reward imbalance and learning engagement

The effort–reward imbalance model (Siegrist, 1996) posits that when individuals perceive that their level of effort is higher than their level of reward, they will have a sense of imbalance, excessively deny themselves, experience a lower level of “homeostasis,” feel a sense of hopelessness for the future, lose confidence in the future, and experience strong negative emotions and sustained stress responses (Zhu and Tao, 2022). As a typical work stress model for studying psychological problems, the imbalance model is widely used in the field of professional work at home and abroad. With the deepening and transfer of related research, this theory has been applied effectively to the field of education, including the two dimensions of effort and reward (Wege et al., 2017). Learning investment is the time and energy that students spend on learning; learning return refers to the outcomes (achievement, rewards, etc.) of learning activities. When students are in a state of high effort and low return for a long time, they are more prone to a sense of effort–reward imbalance. Individuals who are in a state of long-term effort–reward imbalance experience an increase in negative emotions, low self-evaluation and emotional exhaustion (Schulz et al., 2009; Fukuda et al., 2010; Wang et al., 2015), which in turn exacerbates the degree of academic burnout (Wang et al., 2022). Moreover, academic burnout can significantly negatively predict students' learning engagement (Duan and Li, 2008; Zhang et al., 2009; Yan et al., 2018). Therefore, Hypothesis 1 posits that an effort–reward imbalance may negatively predict learning engagement.

### 1.2. Mediating role of learned helplessness

Learned helplessness was originally a psychological concept proposed by Seligman's empirical research with animals (Seligman and Maier, 1967; Yan et al., 2014). Later, further research revealed that humans also exhibit learned helplessness, which leads to various forms of negative repercussions, such as low achievement motivation, low self-awareness, negative emotions, and low self-efficacy (Hiroto, 1974; Miller and Seligman, 1975; Sedek et al., 1993; Boichuk et al., 2014). Learned helplessness in learning refers to the fact that students are unable to succeed under the influence of many factors. In the long run, students' attitudes toward learning become increasingly incorrect, and even negative psychological emotions appear (Yu, 2023). Therefore, learned helplessness can have a negative impact on learning engagement (He and Zhou, 2022; Liu et al., 2023). The theory of learned helplessness holds that external feedback is closely related to individual's learned helplessness behavior and external negative evaluation or negative feedback can lead to negative pessimism and negative attribution style, and ultimately lead to learned helplessness (Abramson et al., 1978; Dweck et al., 1978). When students would not earn the satisfactory reward after they spend a considerable amount of time and energy in learning, their enthusiasm for learning will be reduced, and thus facilitating negative emotions such as pessimism, anxiety, reluctance, burnout and frustration during learning (Braun et al., 2010; Wang et al., 2022). In addition, these symptoms of pessimism, anxiety, reluctance, burnout and frustration during learning are the outward manifestations of learned helplessness. Therefore, according to the theory of learned helplessness, the negative external feedback of the effort-reward imbalance can possibly lead to learned helplessness for students when students experience the effort-reward imbalance. In the other words, the effort-reward imbalance can lead to learned helplessness for students. The emergence of learned helplessness makes students have no enthusiasm for learning and easily abandon lessons, thus reducing their investment and efficiency in learning (Johnson, 1980; Goodall, 2015; Sun, 2022). Therefore, Hypothesis 2 posits that learned helplessness can negatively predict learning engagement and mediate the relationship between effort–reward imbalance and learning engagement.

### 1.3. Moderating role of student social support

Social support, a term formally proposed in the 1970s, refers to social contact that can reduce the psychological stress response, relieve mental stress, and improve social adaptation; this term mainly refers to family members, relatives and friends, colleagues, groups and organizations (Zhao and Wang, 2016). The academic community usually divides social support into two categories: actual support, which is the action taken by others to provide help to the focal person; and perceived social support, which is the cognitive evaluation of having a reliable connection with others (Barrera, 1986). The buffer model posits that social support is a protective mechanism that can alleviate the negative impact of negative events on individuals (Wolff et al., 2014). Several empirical studies have shown that high social support can buffer individuals' negative emotions (Feng and Liu, 2021; Lv and Hu, 2023), change individuals' perceptions of negative life events (Li and Jin, 2014), increase their positive self-cognition and psychological capital (Yarcheski and Mahon, 2014) and improve their mental health (Lv and Hu, 2023); ultimately, individuals can actively respond to the difficulties they experience (Zhu and Tao, 2022). Therefore, these studies strongly support the predictions of the buffer model on the effects of social support. Additionally, individuals who can mobilize social support in the face of negative events tend to experience a weaker impact; that is, social support can protect against the impact of negative events (Yu et al., 2021). As a specific negative event, an effort–reward imbalance can predict students' learned helplessness (Abramson et al., 1978); however, according to the buffer model of social support, it can also be deduced that if students experience a high level of social support from their parents, teachers or friends, even while experiencing an effort–reward imbalance, the impact of learned helplessness will be mitigated by social support. Thus, this approach can decrease the impact of effort–reward imbalance on students' learning engagement. Therefore, Hypothesis 3 posits that social support may moderate the relationship between effort–reward imbalance and learned helplessness.

### 1.4. The present study

**Figure 1:**
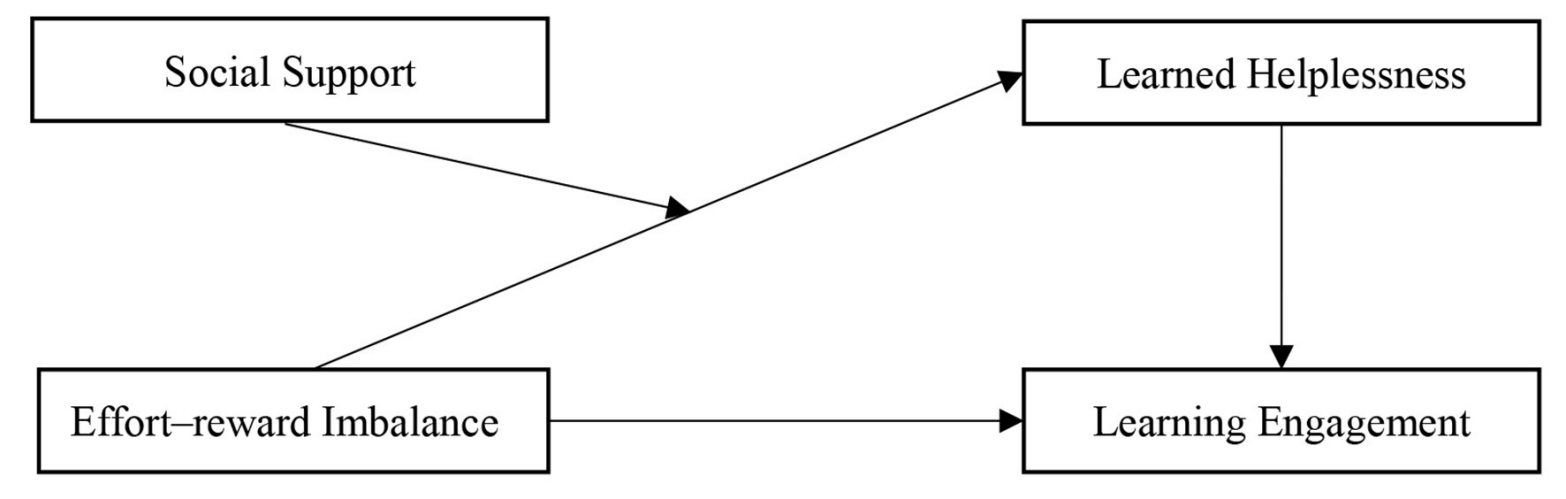
Moderated mediation model diagram.

Effort–reward imbalance is an unavoidable experience for most students, and it can influence student learning engagement. Few studies have investigated how effort–reward imbalance influences students' learning engagement from the perspective of learned helplessness and whether social support can buffer the negative impact of effort–reward imbalance on learned helplessness and thus reduce its impact on students' learning engagement. Based on the effort–reward imbalance model, the buffer model of social support and the results of previous studies, we constructed the following moderated mediation model (Figure 1) to explain the relationships among effort–reward imbalance, learning engagement, learned helplessness and social support, and thus, we revealed how effort–reward imbalance influences students' learning engagement from the dimensions of learned helplessness and social support. This study can also help to test and expand existing theories about effort–reward imbalance and learning engagement and can provide a valuable reference for improving students' learning engagement in education.

## 2 Methods

### 2.1 Participants

A total of 796 students, including 376 male students (47.24%) and 420 female students (52.76%), were selected via convenience sampling from different cities and schools to be participants in the present study. We calculated descriptive statistics for the distribution of students across different grade levels, and the results were as follows: 93 students were in 7th grade (12–13 years old, 11.683%), 264 were in 8th grade (13–14 years old, 33.166%), 5 were in 9th grade (14–15 years old, 0.628%), 175 were in 10th grade (15–16 years old, 21.985%), 167 were in 11th grade (16–17 years old, 20.980%), and 92 were in 12th grade (17–18 years old, 11.558%). In China, students in the 7th to 9th grades attend junior high school, and students in the 10th to 12th grades attend senior high school. After the students and their primary guardians agreed to voluntarily participate in the present survey, the questionnaire was distributed to the participants through an anonymous questionnaire on Questionnaire Star (an internet platform in China). Informed consent was obtained prior to administering the questionnaires, and the research protocol was approved by the medical ethics committee of Huzhou University.

### 2.2 Measures

#### 2.2.1 Effort-reward imbalance for learning scale

This study used the Effort–Reward Imbalance Scale compiled by Fukuda et al. (2010), and the Chinese version as revised by Chu et al. (2015), to measure students' effort–reward imbalance. The scale included two subscales: effort (3 items) and reward (4 items). The scale uses a two-level scoring method, requiring subjects to respond to a given declarative sentence, where 1 means no and 2 means yes. Example statements included “When I study in school or class, I often have to stop because of the interference of other people” and “I will try to perform well in class.” The effort–reward imbalance for learning (LERI) ratio was used to measure the degree of effort–reward imbalance experienced by the students. The ERI ratio = effort score/(reward score × C), where C is the adjustment coefficient (the ratio of the number of items in the effort dimension to the number of items in the reward dimension) and is generally taken to equal 0.75. The greater the LERI was, the greater the effort–reward imbalance students experienced in learning. The scale has been widely used in related research and was found to have good reliability and validity (Chu et al., 2015; Wang et al., 2022).

#### 2.2.2 Academic engagement scale

This study adopts the learning engagement scale compiled by Schaufeli et al. (2002) and revised by Fang et al. (2008), which includes three dimensions, vitality (6 items), dedication (5 items) and concentration (6 items), for a total of 17 items. Example items include “I'm willing to learn as soon as I get up in the morning” and “I'm strong and motivated when I study.” Items are scored on a 7-point scale, with 1 indicating “never” and 7 indicating “always.” The average score of each dimension represents the level of learning engagement of the students. The higher the score is, the greater the level of learning engagement. The scale has been widely used in related research (Liu et al., 2023; Zhang et al., 2023) and was found to have good reliability and validity. The Cronbach's α coefficient of the scale in this study was 0.966, and the Cronbach's α coefficients of the vitality, dedication, and concentration subscales were 0.926, 0.903, and 0.930, respectively.

#### 2.2.3 Learned helplessness questionnaire

The present study used the Learned Helplessness Questionnaire, which was developed by Zeng (2011) to measure students' leaned helplessness. The Learned Helplessness Questionnaire includes four dimensions—cognition (5 items), emotion (7 items), behavior (5 items) and attribution (3 items)—for a total of 20 questions. Example items include “In learning, I have never experienced a sense of achievement” and “I am often overwhelmed by the difficulties encountered in learning.” Items are scored on a 5-point scale, where 1 indicates “completely inconsistent” and 5 indicates “full compliance.” The higher the score is, the greater the degree of learned helplessness. The scale has been widely used in related research (Wang et al., 2022) and was found to have good reliability and validity. The Cronbach's α coefficient of the scale in this study was 0.954, and the Cronbach's α coefficients of the cognitive, emotional, behavioral, and attributional subscales were 0.885, 0.847, 0.852, and 0.803, respectively.

#### 2.2.4 Perceived social support scale

This study used the Chinese version of the Perceived Social Support Scale, which was developed by Blumenthal and his co-operators (Blumenthal et al., 1987), and the Chinese version of the Perceived Social Support Scale was revised by Jiang (1999). The scale is a multilevel social support tool for measuring individual self-understanding and includes three dimensions, family support (4 items), friend support (4 items) and other support (4 items), for a total of 12 items. Example items include “Some people (teachers, relatives, classmates) will appear beside me when I encounter problems,” and “My family is willing to help me make various decisions.” Items are scored on a 7-point scale, where 1 indicates “extremely disagree” and 7 indicates “extremely agree.” The total score reflects the level of social support perceived by individuals. The greater the score was, the more social support the students felt. The scale has been widely used in related research (Feng et al., 2016; Zuo, 2022) and was found to have good reliability and validity. The Cronbach's α coefficient of the scale in this study was 0.91, and the Cronbach's α coefficients of the family support, friend support, and other support subscales were 0.883, 0.897, and 0.871, respectively.

### 2.3 Procedure

Students from 6 middle and high schools in Huzhou city, Zhejiang Province, in China were selected as the main participants by means of convenience sampling. All the questionnaires were edited using Questionnaire Star (an online platform in China dedicated to producing and distributing various questionnaires, which is widely used by scholars in scientific research). From May 6, 2023, to May 21, 2023, we distributed the edited questionnaires to students through the Questionnaire Star platform. We also shared questionnaires on WeChat and Tencent QQ (these communication tools have been widely used in China) to help a wider range of middle school students participate in this survey. Before the survey started, all participants and their principal guardians were given an informed consent form and agreed to participate in the survey voluntarily. All participants were then encouraged to carefully complete all of the edited questionnaires online. After the survey was completed, all of the participants' data were statistically analyzed via appropriate methods. When the questionnaire was completed on the Questionnaire Star platform, only the participants could successfully submit all the questions. Therefore, there are no missing values in this survey.

### 2.4 Design

Based on the effort–reward imbalance model, theory of learned helplessness, the buffer model of social support and the results of previous studies we deduced a moderated mediation model to explain the relation among effort-reward imbalance, learned helplessness, learning engagement and social support. In order to verify the rationality of the model, we aim to use the Effort-reward Imbalance Scale, the Learning Engagement Scale, the Learned Helplessness Questionnaire and the Perceived Social Support Scale to measure students' effort-reward imbalance level, learned helplessness level, perceived social support and learning engagement respectively. After collecting the data, we use appropriate statistical methods to analyse the collected data and verify the deduced moderated mediation model.

### 2.5 Data analysis

SPSS 26.0 was used to conduct a common method deviation test, descriptive statistical analysis and correlation analysis of each variable. The SPSS macro program PROCESS plug-in was used to test the mediating effect of learned helplessness and the moderating effect of social support on the relationship between effort–reward imbalance and learning engagement.

## 3 Results

### 3.1 Common method bias test

Considering that all the questionnaires were self-reported, common method bias may have occurred in the program. For excluding the influence of common method bias on the credibility of the research results, the Harman single-factor test was performed (Podsakoff et al., 2003). Exploratory factor analysis without rotation was also conducted for all items of the Effort-Reward Imbalance Scale, the Learning Engagement Scale, the Learning Heights Questionnaire and the Perceived Social Support Scale. The results of unrotated factor analysis showed that the eigenvalues of nine factors were >1, and the variation explained by the first factor was 32.81%, which is less than the critical value of 40% (Zhou and Long, 2004). Therefore, there was no serious degree of common method bias in this study.

### 3.2 Descriptive statistics and correlation analysis of variables

Table 1 lists the mean, standard deviation and correlation matrix of each variable. The results showed that there were significant correlations between effort–reward imbalance, learning engagement, learned helplessness and social support. The effort–reward imbalance rate was negatively correlated with learning engagement and social support and positively correlated with learned helplessness. Social support was positively correlated with learning engagement, and learned helplessness was negatively correlated with learning engagement. Social support was negatively correlated with learned helplessness, which indicates that it is suitable for further mediating effect analysis.

**Table 1:** Descriptive statistics and correlation analysis (n = 796).

	**M ±SD**	**1**	**2**	**3**	**4**	**5**
1. Grade	3.420 ± 1.651	1				
2. Effort–reward imbalance	1.187 ± 0.304	0.017	1			
3. Learning engagement	3.856 ± 1.165	−0.008	−0.221^**^	1		
4. Learned helplessness	2.402 ± 0.814	−0.024	0.268^**^	−0.458^**^	1	
5. Social support	4.790 ± 1.007	−0.033	−0.314^**^	0.459^**^	−0.336^**^	1

^**^p < 0.01.

In order to avoid the synonymous situation of variables, it is necessary to ensure the rationality and feasibility of the results through multicollinearity test. The multicollinearity problem is determined by the value of the variance inflation factor (Reuben and David, 1986). Through SPSS26.0, the data analysis shows that the variance inflation factor value of each variable does not exceed 2 and far < 10 (Table 2), indicating that there is no serious multicollinearity problem, and the regression model test can be carried out (Gao, 2000).

**Table 2:** Multicollinearity (n = 796).

	**Non-standardized coefficient**		**Standardized coefficient**	***t***	***p***	**VIF**
	**B**	**Standard error**	**Beta**			
Constant	3.258	0.289		11.285	0.000	
Effort–reward imbalance	−0.095	0.121	−0.025	−0.787	0.431	1.15
Learned helplessness	−0.484	0.045	−0.338	−10.633	0.000	1.17
Social support	0.391	0.037	0.338	10.478	0.000	1.20

### 3.3 Mediating role of learned helplessness

Using the model 4 in PROCESS (Hayes, 2017), bootstrap samples were repeatedly selected 5000 times for a simple mediation effect test (Table 3). After controlling for gender and grade, the results showed that effort–reward imbalance significantly positively predicted learned helplessness (β = 0.709, *t* = 7.75, *p* < 0.001; 95% CI = 0.5295–0.8885) and significantly negatively predicted learning engagement (β = −0.8419, *t* = −6.33, *p* < 0.001; 95% CI = −1.1029 to −0.5809). Hypothesis 1 posits that an effort–reward imbalance may negatively predict learning engagement. Simple mediation effect test showed that the effort-reward imbalance has a negative impact on learning engagement. The result was consistent to the prediction of the hypothesis 1, therefore the hypothesis 1 is confirmed.

**Table 3:** Mediation model test of learned helplessness.

**Result variables**	**Predictors**	**R**	**R^2^**	**F**	***B***	**LLCI**	**ULCI**	***t***
Learned helplessness	Effort–reward imbalance	0.2805	0.0787	22.5391	0.7090	0.5295	0.8885	7.7527^***^
	Gender				0.1278	0.0185	0.2371	2.2962
	Grade				−0.0161	−0.0491	0.0170	−0.9557
Learning engagement	Effort–reward imbalance	0.2251	0.0507	14.0928	−0.8419	−1.1029	−0.5809	−6.3311^***^
	Gender				−0.0954	−0.2543	0.0635	−1.1786
	Grade				−0.0013	−0.0493	0.0467	−0.0531
Learning engagement	Effort–reward imbalance			55.8282	−0.4063	−0.6519	−0.1607	−3.2479^**^
	Learned helplessness	0.4692	0.2202		−0.6144	−0.7064	−0.5224	−13.1115^***^
	Gender				−0.0169	−0.1615	0.1277	−0.2290
	Grade				−0.0112	−0.0548	0.0324	−0.5035

^**^ denotes *p* < 0.01; ^***^ denotes *p* < 0.001.

After incorporating the mediating variable learned helplessness, the negative predictive effect of effort–reward imbalance on learning engagement was still significant (β = −0.4063, *t* = −3.2479, *p* < 0.01, 95% CI = −0.6519 to −0.1607), and the negative predictive effect of learned helplessness on learning engagement was significant (β = −0.6144, *t* = −13.1115, *p* < 0.001), 95% CI = −0.7064 to −0.5224). The positive predictive effect of effort–reward imbalance on learned helplessness was significant (β = 0.7090, *t* = 7.7527, *p* < 0.001, 95% CI = 0.5295 to 0.8885). The above results preliminarily indicated that learned helplessness mediates the relationship between effort–reward imbalance and learning engagement.

**Figure 2:**
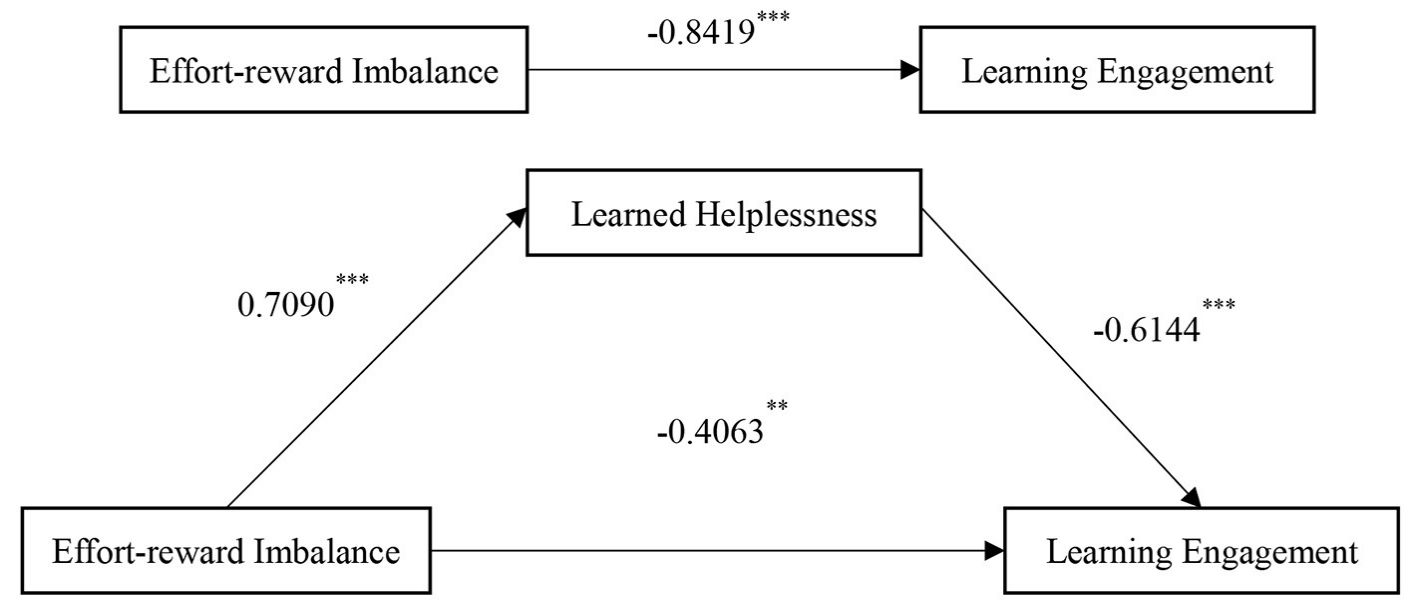
The mediating model of learned helplessness. ^**^ denotes *p* < 0.01; ^***^ denotes *p* < 0.001.

The effort–reward imbalance rate was the independent variable, learning engagement was the dependent variable, and learned helplessness was the mediating variable. The relationship between effort–reward imbalance and learning engagement was investigated, including both the direct effect and the indirect effect, with learned helplessness as the mediating variable. The results showed that the direct effect of the effort–reward imbalance ratio on learning engagement was −0.4063 (95% CI = −0.6519 to −0.1607), and the indirect effect mediated by learned helplessness was −0.4356 (95% CI = −0.5823 to −0.3009). The above data reveal that the confidence interval of the direct effect does not contain zero, and the confidence interval of the indirect effect also does not contain zero. This finding showed that the indirect effect of effort–reward imbalance on learning engagement through learned helplessness was statistically significant; even after controlling for learned helplessness, the direct effect of effort–reward imbalance on learning engagement was still significant. Therefore, learned helplessness partially mediates the relationship between effort–reward imbalance and learning engagement. Hypothesis 2 is confirmed, and a model diagram of effort–reward imbalance → learned helplessness → learning engagement is obtained (Table 4 and Figure 2).

**Table 4:** Analysis of the mediating effect of learned helplessness.

	**Effect**	**SE**	***p***	**95% CI**		**Relative effect size**
				**LLCI**	**ULCI**	
Total effect	−0.8419	0.1330	0.0000	−1.1029	−0.5809	
Direct effects	−0.4063	0.1251	0.0012	−0.6519	−0.1607	48.26%
Indirect effects	−0.4356	0.0708		−0.5831	−0.3077	51.74%

### 3.4 Moderating role of student social support

In the moderated mediation model test proposed by Wen and Ye (2014), each variable is standardized, and the PROCESS plug-in model 7 is used (Hayes, 2017). Introduction to mediation, moderation, and conditional process analysis: A regression-based approach. New York, NY: Guilford Publications.). Repeated bootstrap samples were used 5000 times to test whether the mediating effect of learned helplessness was moderated by social support. After controlling for grade and gender, the results showed that an effort–reward imbalance negatively predicts learning engagement, and the interaction between effort–reward imbalance and social support was significant (β = 0.2416, *t* = 2.4936, *p* < 0.05; 95% CI = 0.0514 to 0.4317), indicating that social support moderates the effect of an effort–reward imbalance on learned helplessness. Thus, a moderated mediating effect was detected (Table 5).

**Table 5:** Moderated mediating effect test (n = 796).

**Variable**	**Dependent variable: learned helplessness**			**Dependent variable: learning engagement**		
	β	***t***	**95% CI**	β	***t***	**95% CI**
Gender	0.1012	1.8912	(−0.0038, 0.2063)	−0.0169	−0.2290	(−0.1615, 0.1277)
Grade	−0.0143	0.8746	(−0.0462, 0.0177)	−0.0112	−0.5035	(−0.0548, 0.0324)
Effort-reward imbalance	0.5430	5.6574^***^	(0.3546, 0.7314)	−0.4063	−3.2479^**^	(−0.6519, −0.1607)
Social support	−0.2195	−7.8518^***^	(−0.2744, −0.1646)			
Effort-reward imbalance × social support	0.2416	2.4936^*^	(0.0514, 0.4317)			
Learned helplessness				−0.6144	−13.1115^***^	(−0.7064, −0.5224)
R^2^	0.1541			0.2202		
F	28.7870^***^			55.8282^***^		

^***^p < 0.001; ^**^p < 0.01; ^*^p < 0.05.

**Figure 3:**
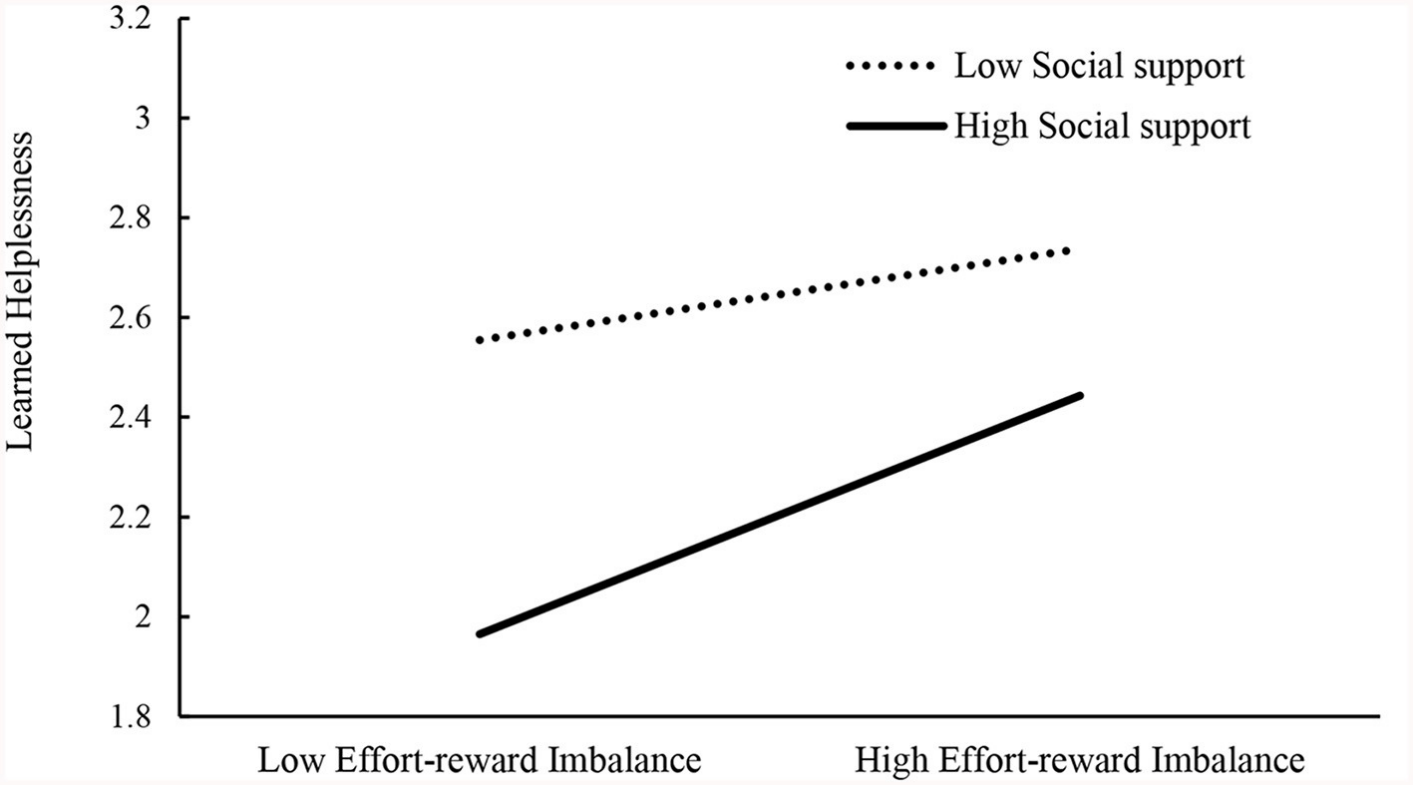
The moderating effect of social support.

To further explore the nature of the interaction between social support and effort–reward imbalance, a simple slope test analysis of the groups with high levels and low levels of social support was performed, and the results showed that for students with low levels of social support, the impact of effort–reward imbalance on learned helplessness was relatively small; however, for students with high levels of social support, the effect of effort–reward imbalance on learned helplessness was more significant (Table 6 and Figure 3). A deepening of effort–reward imbalance has a more significant impact on students with high levels of social support. In situations of low effort–reward imbalance, there is also a substantial disparity in learned helplessness between individuals with high and low levels of social support. However, as the degree of effort–reward imbalance increases, the levels of learned helplessness for individuals with high and low levels of social support gradually converge. Importantly, in situations of low or high effort–reward imbalance, the level of learned helplessness is significantly lower for individuals with high levels of social support than for individuals with low levels of social support. Therefore, based on the aforementioned results, it can be concluded that social support moderates the effect of effort–reward imbalance on learned helplessness; thus, hypothesis 3 holds.

**Table 6:** The moderating effect of social support.

	**Low effort–reward imbalance**	**High effort–reward imbalance**
Low social support	2.5549	2.7371
High social support	1.9654	2.4430

## 4 Discussion

Although effort–reward imbalance is common in the learning process, few studies have examined whether this imbalance affects learning engagement and the underlying mechanism involved. Based on the effort–reward imbalance model and the buffer model of social support used in related research, this study speculates that effort–reward imbalance can negatively predict students' learning engagement, that learned helplessness mediates the relationship between effort–reward imbalance and learning engagement and that social support can moderate the relationship between effort–reward imbalance and learned helplessness.

The results indicate that effort–reward imbalance could negatively predict learning engagement, implying that effort–reward imbalance is one of the important predictors of students' learning engagement. Previous studies have explored the influencing factors and mechanisms of learning engagement in the context of academic emotions, perceived social support, proactive personality traits and academic burnout (Zhang et al., 2016; Bao et al., 2022; Zuo, 2022; Liu, 2023). This study further clarifies the impact of students' experience of effort–reward imbalance on learning engagement, identifies a way to improve students' learning engagement, and enriches the theoretical research on learning engagement.

### 4.1 Mediating role of learned helplessness

Based on previous studies (Seligman and Maier, 1967; He and Zhou, 2022; Liu et al., 2023), we deduced that learned helplessness plays a mediating role between effort–reward imbalance and learning engagement. The results of this study showed that an effort–reward imbalance can directly affect learning engagement and indirectly affect learning engagement through learned helplessness, suggesting that learned helplessness indeed plays a mediating role in the relationship between effort–reward imbalance and learning engagement. This result confirmed our hypothesis. In several previous studies, the authors indicated that negative events can dampen students' learning enthusiasm and reduce their learning engagement (Zhang et al., 2008; Xu, 2022). When students do not earn the rewards they expect after they expend more effort on their studies, they often regard their studies as unsuccessful. In this way, we can regard the effort–reward imbalance as a negative event. Our study showed that effort–reward imbalance influences learning engagement through the partial mediating role of learned helplessness. This result is consistent with the predictions of Wang et al. (2022) study.

Why can helplessness partly mediate the relationship between effort–reward imbalance and learning engagement? On the one hand, the occurrence of an effort–reward imbalance means that the individual is not rewarded for the learning activities. Students who experience effort–reward imbalance are prone to negative emotions such as frustration and anxiety (Linnenbrink-Garcia and Pekrun, 2011; Pekrun and Linnenbrink-Garcia, 2012). In the long run, students' self-confidence will decrease, leading to negative self-suggestions such as “I can't” or “I'm stupid” and doubting their own ability. Thus, these students will experience negative motivational, emotional, cognitive and behavioral states, further cementing the idea that “no matter how hard you try, nothing can be done.” When individuals encounter difficulties, they give up and generate a sense of learned helplessness (Ma, 2004; Lv, 2012). On the other hand, a sense of learned helplessness in learning can have a negative impact on students' future learning, such as reduced learning motivation, passive treatment of learning and obstacles in cognition (i.e., a loss of confidence in oneself can lead to the feeling that knowledge and skills are difficult to master). Furthermore, emotional disorders, feeling annoyed, pessimism, and depressive symptoms can lead to a decrease in the level of learning engagement (Cui, 2012; Zhang et al., 2016; Jiang et al., 2019; Deng et al., 2023; Huang, 2023).

By studying the mediating role of learned helplessness in the relationship between effort–reward imbalance and learning engagement, it can be found that learned helplessness is one of the important mechanisms through which the effort–reward imbalance affects learning engagement. This study clarifies the mechanism through which effort–reward imbalance influences learning engagement and supplies insight on how effort–reward imbalance influences learning engagement through the dimension of learned helplessness. In addition, considering that students often face an effort–reward imbalance in their daily learning, this impact on learning attitudes was not effectively avoided. According to the results of the present study, an effort–reward imbalance can influence students' learning engagement through the mediating role of learned helplessness. These findings also suggest that we should pay attention to and reduce the occurrence of students' effort–reward imbalance as much as possible and prevent the negative impact on student learning.

### 4.2 Moderating role of student social support

This study revealed that social support plays a significant moderating role in the effect of effort-reward imbalance on learned helplessness. High social support can buffer the impact of effort–reward imbalance on learned helplessness. Specifically, an effort–reward imbalance can aggravate students' learned helplessness, but high social support can provide spiritual and material help for individuals and thus buffer the impact on students' learned helplessness. A further simple slope test revealed that the protective effect of social support is more obvious when the effort–reward imbalance is low. The buffer model of social support indicates that social support can alleviate the negative impact of negative feedback (Wolff et al., 2014). This study revealed that social support can buffer the negative influence of an effort–reward imbalance on students' learned helplessness. The results of the present study further verify the buffer model of social support. Moreover, the reverse stress-buffering model indicated that individuals may inhibit the buffering effect of positive psychology on stressful events in a certain stressful environment (Rueger et al., 2016). Previous studies on the effort–reward imbalance have shown that when students have a high imbalance, this imbalance can lead to high levels of negative emotions such as stress and anxiety (Linnenbrink-Garcia and Pekrun, 2011; Pekrun and Linnenbrink-Garcia, 2012). According to the reverse stress-buffering model, it can be deduced that the protective effect of social support on the negative effect of effort–reward imbalance was more effective in the low effort–reward imbalance condition; in contrast, when the effort–reward imbalance students faced was greatest, high levels of stress and anxiety might inhibit the protective effect of social support. The moderating effect of social support showed that the protective effect of social support is more obvious when the effort–reward imbalance is low. This finding is also consistent with the prediction of the reverse stress-buffering model. Therefore, the results of the present study can also verify the reverse stress-buffering model.

The finding of a moderating effect of social support enlightens us that when students have an effort–reward imbalance, parents, teachers and other members of society should provide support, which can improve students' social support level and thus help them alleviate the negative impact of effort–reward imbalance on their studies. Of course, it should be noted that the protective effect of social support cannot be exaggerated because that the efficacy of the protective effect could be decreased as the effort–reward imbalance increases. Therefore, to improve students' learning engagement more effectively, on the one hand, we should provide support to buffer the negative influence caused by the effort–reward imbalance; on the other hand, we should also pay attention to students' sense of effort–reward imbalance and reduce the imbalance as much as possible. Only in this way can we more effectively prevent the negative impact on student learning.

## 5 Limitations and future research

The present study examined how effort–reward imbalance influences the learning engagement of students from the perspective of learned helplessness and social support based on the effort–reward imbalance model and the buffer model of social support. The results showed that effort–reward imbalance influences students' learning engagement partly through decreasing their learned helplessness; moreover, social support can buffer the negative impact of effort–reward imbalance on learned helplessness and thus buffer the impact on students' learning engagement. The results of this study enrich the research on the relationship between the effort–reward imbalance and learning engagement and fill the gap in explaining how this imbalance influences learning engagement from the perspective of learned helplessness. This study has several limitations that need to be further studied. First, cross-sectional data were collected to fit the proposed model on how effort–reward imbalance influences students' learning engagement. Although the results of the study can preliminarily reveal how effort–reward imbalance influences learning engagement, they are not as convincing as longitudinal data. Subsequent studies can further explore the causal relationships among variables using longitudinal data or design related experiments, which would be more convincing for revealing the influence of effort–reward imbalance on learning engagement. Second, the present study testifies only to the influence of effort–reward imbalance on learning engagement from the dimensions of learned helplessness and social support. Subsequent studies should reveal the influence of the effort–reward imbalance of learning engagement from other dimensions. Finally, the data were collected online by convenient sampling method to test the hypothesis model in this study. Although the results well fit our hypothesis, the external validity of the results is limited because of the limitation of the convenient sampling method. Therefore whether the result of the present study can also apply to a larger numbers of other middle school students was needed further testify. And thus further studies can validate and extend the result of the present study on a larger scale, and further increasing the external validity of the present study to better guide our education.

## 6 Conclusion

The study intends to examine the predictive effect of effort-reward imbalance on learning engagement, and the mediating mechanism of learned helplessness and the moderating mechanism of social support in the relation between effort-reward imbalance and learning engagement. According to the results of the present study, it can be concluded that: (1) Effort–reward imbalance is an important predictor of students' learning engagement. (2) As a negative and special psychological state, learned helplessness mediates the relationship between students' effort–reward imbalance and learning engagement. (3) Social support moderates the relationship between effort–reward imbalance and learned helplessness. High levels of social support can buffer the impact of effort–reward imbalance on learned helplessness. The present study further reveal the mediating mechanism of learned helplessness in the relation between effort-reward imbalance and learning engagement and the moderating effect of social support. The result implies that educators should pay attention to and reduce the occurrence of students ' effort-reward imbalance as much as possible in the teaching process, so as to prevent negative impact on students' learning engagement. In addition, educators can also provide support for students from multiple perspectives to improve their social support level, so as to help them reduce the negative impact of effort-reward imbalance on learning.

## Data availability statement

The raw data supporting the conclusions of this article will be made available by the authors, without undue reservation.

## Ethics statement

The studies were approved by the Ethics Committee of Huzhou University. The studies were conducted in accordance with the local legislation and institutional requirements. Written informed consent for participation in this study was provided by the participants' legal guardians/next of kin.

## Author contributions

SL: Data curation, Investigation, Writing – original draft. YW: Writing – original draft. WH: Writing – review & editing. YC: Conceptualization, Writing – review & editing. QW: Conceptualization, Writing – review & editing.
